# HAM/TSP introduction: systematic review of HAM/TSP clinical trials

**DOI:** 10.1186/1742-4690-11-S1-O26

**Published:** 2014-01-07

**Authors:** Rebecca Anderson, Fabiola Martin

**Affiliations:** 1 Department of Biology, University of York, York, UK; 2 Centre for Immunology and Infection, Department of Biology, Hull and York Medical School, University of York, York, UK

## Background

HTLV-1 Associated Myelopathy/Tropical Spastic Paraparesis (HAM/TSP) is a chronic disabling disease caused by the retrovirus HTLV-1. It is a horrible, chronic, progressing disease causing weakness of lower limbs, bladder, bowel and erectile dysfunction, as well as pain in the lower back and legs. HAM/TSP is associated with significantly higher mortality and morbidity than the general population. Currently there is no cure or antiviral treatment for HTLV-1 and no internationally agreed treatment for patients with HAM/TSP. We had two aims: to identify the level of evidence of current medicinal treatment strategies available to patients and clinicians and to identify drugs that should be pursued for future clinical trials.

## Methods

Using the PICOS search strategy and inclusion (adults+HAM/TSP+drug treatment+ english), exclusion (sample size <5, duration <2 weeks, <1985, in vitro, case studies) criteria all papers identified in PubMed were analysed. Each paper was scored individually using a modified quality assessment questionnaire (scores 0-23).

## Results

24/3012 manuscripts were included in the final analysis. The most commonly tested drugs were corticosteroids (9%) and interferon (29%) but only two were randomised controlled trials (RCT) scoring 20. One was a double blind placebo RCT (DBRCT) of zidovudine and lamivudine. The other interferon DBRCT dose finding trial. None of the trials were comparable due to very different outcome measures and patient groups. Therefore the level of evidence was 4 to 5 only.

## Conclusions

Most HAM/TSP treatment studies are either observational or proof- of-concept studies where outcomes cannot be generalised. Both corticosteroids and interferon need to be tested further in clinical trials with 90% powered sample sizes, either against placebo, or best local standard of care or head to head against each other.

